# Cultural Image of Older People during the COVID-19 Pandemic

**DOI:** 10.3390/ijerph192214644

**Published:** 2022-11-08

**Authors:** Eneritz Jiménez-Etxebarria, Joana Jaureguizar Alboniga-Mayor, Elena Bernaras Iturrioz

**Affiliations:** 1 Department of Developmental and Educational Psychology, University of Basque Country UPV/EHU, 48940 Leioa, Spain; 2 Department of Developmental and Educational Psychology, University of Basque Country UPV/EHU, 20018 Donostia, Spain

**Keywords:** pandemic, older people, social imaginary

## Abstract

The COVID-19 pandemic has made evident the exclusion to which older people may be subjected for reasons of age. This study delves into the cultural image of older adults during the pandemic from the perspective of people between 60 and 81 years of age. Through a qualitative methodology, the voices of 37 people have been collected through in-depth interviews. Two main themes are derived from the inductive analysis: on the one hand, the devaluation of older people, and on the other hand, the positive image of the older population as older and valid. We conclude that people over 60 years of age in the Basque Country denounce the stigma of low capacity attributed to the older population during the pandemic. They reject the signs of age-based overprotection manifested during the pandemic and highlight the vital experience by which older people could be considered referents in situations of social crisis. They reflect on the initiatives necessary to improve the cultural image of the older population and point out the opportunities for active ageing, education based on values and intergenerational relationships.

## 1. Introduction

The COVID-19 pandemic has demonstrated the exclusion to which older people may be subjected for reasons of age [1,2]. Public information and social behaviour have been very focused on this group, often giving into ageist approaches [3,4]. The association between the concept of “older person” and ideas such as vulnerability, loss and lack of attention has been predominant in the context during the COVID-19 pandemic [5]. A study at the beginning of the pandemic found that more than half of a Spanish sample of 840 people considered that older adults had poorer information processing skills, should receive more recommendations, were more afraid than other age groups and generated a greater burden on the health system [3]. This corresponds to an already existing negative and stereotyped image of older people that has been brought to the forefront in the context of COVID-19 [2].

Since the start of the pandemic worldwide, emergency policy and health measures were adopted to protect the population, and alerts were issued about the most vulnerable groups, including older adults, regarding the health risks derived from infection with COVID-19 [6,7]. This differentiation between the general population and risk groups, which in the case of Spain has entailed selective preventive measures for certain social collectives, has led to the study of discriminatory attitudes in diverse fields. It was evident that the language used during the pandemic has been exclusionary in public contexts, with the use of terms such as “old” and “grandparents” in reference to the older population [4,5,8]. Likewise, social or health policies have also been discriminatory and resulted in behaviours such as longer-term confinement for older people or age-based prioritisation criteria for health care [2,9,10]. Distinctions between young and old imply intergenerational conflict [11]. Thus, the older people’s condition has been categorised as vulnerable and fragile, confirming typical stereotypes of the ageism phenomenon [12]. 

This negative cultural image has been perceived by older people from different countries who have observed discriminatory behaviour in the contexts of health, media and daily activities [13]. They stated that the image of an active older person was abandoned and replaced by a stigma that prevailed during the pandemic of frail, older persons in need of help [13]. In this study, the use of stigmatisation, paternalism and the search for scapegoats represented older people as a homogeneous group. According to the stereotype content model (SCM) [14], the display of a paternalistic attitude towards a group, like that of the older population perceived as warm, but not very competent, is common [14,15]. 

The negative consequences of ageism for people and society have been documented historically [16,17,18] and in scientific evidence generated during the COVID-19 pandemic [3,13,19,20]. Though this phenomenon can affect older people in general, not all seem to be equally vulnerable to the manifestation of ageism. Among the findings in literature related to the COVID-19 pandemic, we highlight a review according to which a positive identity towards the age group, a positive experience of the present and future ageing process itself, the acceptance of one’s own body and flexibility in establishing personal goals were protective factors against the influence of ageist manifestations [20]. In addition, mediating variables have been identified, such as a person’s physiological state, self-efficacy and support network [10]. On the other hand, adopting a compliant attitude in the face of stereotyped information about the older population could lead to feeling older or expressing thoughts coherent with ageist content [20]. According to this most recent study, attitudes towards ageist manifestations could vary, from acceptance to rejection [2]. This evidence is an opportunity to intervene in reducing the impact of ageism on people, which has shown favourable results in previous literature [20]. 

Given the foregoing, this study aims to delve deeper into the perception of people over 60 years of age in relation to the social imaginary of older adults during the COVID-19 pandemic. 

## 2. Materials and Methods

The study was based on a qualitative approach. We sought to give prominence to the voice of older people so that they could describe their point of view and feelings in relation to a psychosocial phenomenon that can directly or indirectly affect them [17,21]. The conversations took place using the in-depth interview technique, at all times with sensitivity in the context of the interpersonal relationship. A sequential design and thematic analysis were applied. The narratives were coded and compared using the NVivo-12 word processing software, which allows data processing and analysis. 

There were 37 participants (29 women, 8 men), residents of the Basque Country. The level of education was: 29.7% (*n* = 11) primary education, 18.9% (*n* = 9) high school or vocational training, and 45.9% (*n* = 17) higher education. The homogeneity criteria correspond to an age between 60 and 81 years.

The interviews were numbered with an alphanumeric code to guarantee anonymity. First, the interview number was used, with the acronym (EN), the number assigned to each participant and their gender: woman (M), man (H).

To discover the participants’ points of view as regards the focus of this study, a psychologist—a member of the research team—contacted them by phone and conducted in-depth interviews. The initial contact was started using convenience sampling. We contacted a person who is an active member of an association for older people. After this, the snowball technique was used to contact more people. We asked participants to suggest people who were older than 60 years of age who might be interested in participating and were different from themselves. Two people were excluded because of hearing problems. In the interviews, a horizontal dialogue was favoured so that the participants could respond freely to the question: “What image do you think society has of older people and what image has been given of older people during the pandemic?”. Clarifying questions were asked in a personalised manner to understand the narratives. For example, interviewee EN6M74: “When referring to our vision, do you mean the image of society at large?”. Or interviewee EN27H71: “When mentioning news disseminated by the media, can you give me an example?”. Closed-ended questions were also used to collect sociodemographic data, sex, age and level of education. The interviews lasted an average of half an hour and were recorded and transcribed. The transcription was carried out by one of the team’s researchers, and then translated into English by a native translator. Ethical regulations were respected at all times and the Ethics Committee of the University of the Basque Country UPV/EHU had approved the study (TI0139).

### Data Analysis

**Figure 1:**
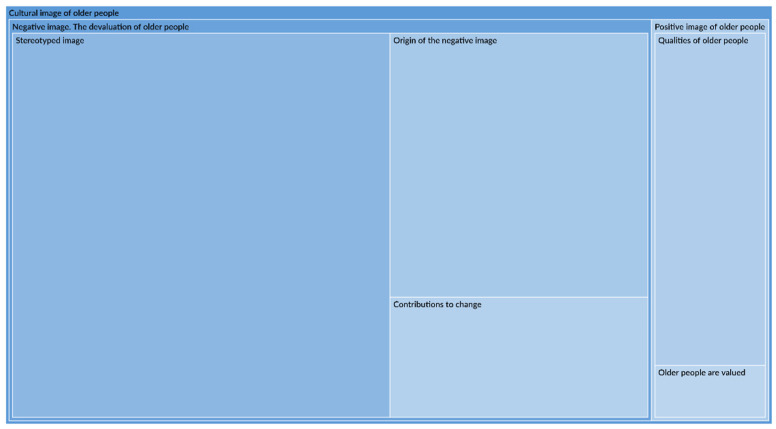
Hierarchy chart of codes. Cultural image of older people.

The participants’ discourse was analysed using an inductive method and their main interests were identified in relation to the cultural image of older people during the COVID-19 pandemic. For the construction of knowledge, a general analysis of the discourses was done. The themes that emerged were refined according to the series of phases established by the grounded theory methodology [22]. The units of meaning were manually codified and subsequently cleaned and classified into theoretical categories that emerged, resulting in a categorical system with the central themes of the cultural image of older adults and the treatment of the older population. To answer this study’s research question, the subcategories of the cultural image of older adults were explored in depth, resulting in two central categories (Figure 1). First, the devaluation of older people, with the subcategories: contributions to change, stereotyped image, origin of the negative image, reaction to discriminatory manifestations. Second, the positive image category of older people, with the representative subcategories: qualities of older people and valuation of older people. The analysis concentrated on the context of the COVID-19 pandemic and also included units of data that did not refer only to COVID-19.

## 3. Results

When the participants describe the cultural image of older people, we observe two opposing attitudes: on the one hand, the devaluation of older people, a main theme that, in turn, includes four other thematic subcategories; on the other hand, the description of the older population as older and valid, which, in turn, encompasses two subcategories.

### 3.1. Negative Image: The Devaluation of Older People

The majority of the participants, 36 out of 37, perceive negative aspects towards older people, as described below.

#### 3.1.1. Stereotyped Image of Older People

We have observed that only two discourses directly allude to the term age stereotypes; however, a detailed analysis leads us to identify that of the 37 participants, 35 narrate the society’s stereotyped vision of the group of older people. “An older person attends to you and you dislike this, you always blame it on the fact that the person is old, as a characteristic that differentiates that person from others” (EN29M69). This perspective reveals with concern that older people are being relegated. It is a common feeling prior to the pandemic: “You start noticing, for example, that you enter a store, as an example, they see you and look at you, but do not take you into consideration, they feel as if this lady is not going to buy here” (EN24MC64). They say this also occurred during the pandemic. As proof of this, they question the condition of older adults who are institutionalised in nursing homes: “The image has not been positive, especially as regards nursing homes (…) the COVID has stirred consciences” (EN17H69) and they mention some health measures adopted according to age-based criteria that reflect the lesser value given to people when they reach an advanced age: “In these times no treatment is going to be given because, given their age, it’s time to die” (EN19H64).

We also collected negative qualifiers used when describing the older population, where adjectives prevail in reference to their state of dependency and vulnerability. “Older, a limited person, people with difficulties, an asocial person. (…) I think this is a mistake (…) like someone vulnerable, someone incapable of self-management” (EN9M62). A belief that promotes behaviours such as overprotection of the older population as shown during the pandemic, regardless of its capacity for autonomy: “As if they were incapable of doing things on their own (…) There may be exceptions, but other cases (…) we can manage well, we don’t need that -let’s call it- overprotectiveness” (EN21M69). 

Likewise, we identified another opinion that all older people are viewed as having the same characteristics, especially with attributions that devaluate them: “Too much emphasis is being placed on older people as fools. And an older person is not a fool” (EN11M63).

A participant who collaborates in a work group on ageing shares his knowledge and affirms the followings:

The social imaginary of the older population has not evolved. (…) it does not fit the reality of older people. We have an important flaw, we define things strictly based on age, chronological age, without considering other types of things, that is called ageism, age-based exclusion (…) the other mistake (…) is that it seems that we are a homogeneous group. (…) If you consider that someone is older as of the age of 60 years, between 65 and 90, within such a disparate and wide chronological age range we are in all types of very different physical conditions, you cannot lump us all together. (EN30H81).

Another issue of concern is that older people are not productive because they are not economically active, a perception to which 7 of the 37 participants also add that they are considered major consumers of social and economic resources. A participant expresses it with feelings charged with negative emotion.

The spoils of society. We were worth nothing. The only thing we did, according to the general opinion or partial opinion of some people, was consume State funds, the public pension funds (…) exhausting the State’s assets (EN6M74).

In the following participant’s discourse, we see that this perception extends across the entire COVID-19 pandemic.

The hospitals were full of patients with coronavirus, and a selection had to made as to who they were going to attend to and who not. And one of the reasons given was that these people are no longer productive (EN24M64).

Along the same lines, a concern arises around the perception of burden associated with older people. The origin of this burden for some people is found in the loss of physical and/or mental capacities. “The pace of life is much calmer, slower and as long as you are well, then there is no problem. The problem is when you start being a bother (…) at that moment, society, well, assumes the burden” (EN10M65). However, there are those who say that the fact of being older in itself makes people a burden in the eyes of society: “It seems that once they are very old, we are a nuisance. (…) there are people who (think that) where can they be better than in a nursing home?” (EN28M81). One person gives the example of a politician’s opinion made public: “The governor of Texas said that older people should not complain about dying if they saved the United States from a recession with their death” (EN8H69), thereby implying that they pose an economic burden to the country. 

#### 3.1.2. Origin of the Negative Image towards Older People

The participants reflect on the causes of a reality that 13 of the 37 interviewees openly reject and agree, on the one hand, as to the role played by the media, especially television, in disseminating an opinion about older people. They narrate examples of the use of generalisations and the transmission of a biased image of the older population.

The radio stations invite us to go talk about older people, about active ageing and such, and then other programs that have nothing to do with it are constantly ridden with clichés. (…) Every time there is news about the older population, the image they display is of a person using a cane, sitting on a bench or alone. (EN29M69).

Regarding the pandemic, they mention how much people talked about the disease and its risks for older people: “they are giving the idea that older people (…) can contract the illness or become sick much more than young people” (EN14H70) or “it has been magnified, there are people who have been quite alarmed (…) have felt afraid because of the news” (EN39M60).

Additionally, the origin of the negative social imaginary is attributed to cultural changes according to which values such as respect have been devalued. It is quite common to feel that there is great disrespect for older people; in some cases, they say they have never seen such a lack of respect as there is now.

What the older adults said was sacred (…) but it would be necessary (…) to instil in the youth that respect should never be lost, and that the older adults have also been young like them and that they, God willing and are allowed, will also reach old age (EN25M79).

We also find mentions of disinterestedness in the value of experience. It seems that the wisdom provided by lifetime experiences is not valued: “Older people are no longer wisdom, that this is why we have technology, and the experiences and tales that they share are dismissed, which are very valuable, because it is wisdom” (EN10M65). 

They express mourning for the knowledge that is being lost and criticise the appraisement of other values such as youth: “So it seems to me that a lot is being missed out on, a lot of experience and very much so. But well, I think that now (…) what is preached somehow is eternal youth” (EN19M64). 

Additionally, social crises, such as the context of the pandemic, have precipitated changes in the imaginary of older people. In this regard, they repeat the dissemination of an image of people’s vulnerability: “They want us to believe the image that older people are in a precarious condition, much more exposed than younger people” (EN14H70).

To a lesser extent, 8 of the 37 participants associate the origin with the observed intergenerational differences, and some people consider that the negative view extends mostly across people of later generations.

We persons who are already at this age, like me, that’s why we have a more respectful image of older people. We know we must take care of them, all of that. (…) However, young people, I don’t know, perhaps it’s because of their age. I think they are unaware of an older person’s importance, their merit or the value that should be given to an older person (EN11M63).

#### 3.1.3. Proposals to Promote a Favourable Image of the Older Population

In this section, 15 of the 37 participants reflect on aspects that could improve the image of the elderly.

Participants consider that the activity a person can perform is one aspect that determines the way they are viewed. Therefore, the need to generate opportunities so that older people can carry out different types of activities is detected: “At least whoever wants to work should be allowed to work longer” (EN8H69). “You have more time available (…) and you can make it available to society, being socially active, (…) from recreational activities to caretaking or whichever type of response you wish to give” (EN30H81). 

Another aspect deserving attention is the image that is disseminated in the media of the group of older people. Some aspects that must be improved are the language, references or clichés used when referring to the older population: “through the media, television, radio or newspaper, (…) showing our lives and what we can still contribute to society or at our age” (EN6M74).

Facilitating spaces for intergenerational interaction is also an idea that for some people would entail progress for society: “It seems unnatural to me to try to do it in watertight compartments by age (…) I think that interaction between generations is essential for a society to be strong in reality. This is essential in the transmission from grandparents to grandchildren” (EN9M62).

A man who is an active participant expresses:

I struggle a lot with the university for senior citizens’ experience (…) because it is not organised in such a way that enables an exchange with a common space, young and old together, because they are all there for learning. Some to practice a profession, others for personal enrichment or to complement something that one had not had the opportunity to learn. But they would meet on the bus, in the halls, in the library. There would be an interrelation between the older adults and the young, that would help youth perceive the older population differently. (EN30 H81)

Faced with the devaluation of older people, they emphasise the importance of an education based on values such as respect, “I always tell my children (…) also teach them values, that is the most important thing. A person who has no values is not human. Even if someone’s a master gunsmith, if they have no values, it’s worth nothing” (EN12M68), or in reference to health protocols applied during the pandemic: “You should never do to anyone what you don’t like being done to you, period. But that is education, especially consideration for older people. Nobody has to die” (EN28M81).

### 3.2. Positive Image of Older People: They Are Older and Valid

In 21 of the 37 speeches, we found allusions to the positive aspects with which older people are viewed, as described below.

#### 3.2.1. Qualities of the Older People

The participants reflect on the better condition of older people today, compared to previous times: “people remain younger, more active until older ages” (EN11M63).

We detect above all the value placed on the life experience that people acquire with age: “They are part of our culture and we also learn from their experiences” (EN17M69). That makes them referents in the eyes of some people. The following testimony exemplifies the role that older people can play in situations of confinement and crisis derived from the COVID-19 pandemic: “Their entire existential journey is very, very important to help us cope with or manage this extreme situation we are experiencing. Many of them have lived through the war and the post-war, what confinement means, what curfew means” (EN9M62). 

They also highlight their ability to confront difficult personal or social situations: “Well, in 2008, when the crisis began, how this generation has moved half the country forward” (EN6M74). The following testimony highlights their interest in social issues: “The whole matter of gathering together to demand pensions for retirees, they even attend the Women’s Day demonstrations” (EN35M69).

Others consider that the image may have improved in certain social contexts, such as in the situation of the national economic crisis:

The perception of older people has improved with the crisis (…) because (…) older people have lent a helping hand economically, with all of their children’s problems, they have moved forward (…) helping financially. I think that then, even at the levels of society and media, the older population was valued (EN35M69).

Regarding the interruption of their social work during the confinement because of the pandemic: “Now I suppose that it will continue to be valued, now that we cannot leave the house, I am not a grandmother, but now the grandparents cannot leave the house to take care of the grandchildren” (EN6M74). 

Three people use positive adjectives: responsible, active, calm and generous to describe older people. The positive adjective wise was also identified; however, it has only been used to testify to the wisdom that was traditionally attributed to them: “Old people are no longer wise, in their place we now have technology” (EN10M65). Finally, in the testimonies that highlight a positive image, we identify expressions that are recognised as personal stereotypes.

They’re awesome, the most socially active people that I know, they’re awesomely involved in choirs, dance groups. They are involved, the most generous people, who lend themselves to anything to help or with very prosocial ideas (EN9MS62).

#### 3.2.2. Perception That Older People Are Valued

A minority, 6 of the 37 interviewees, perceive that older people are perceived through a respectful attitude: “I think that people, in general, have respect and some gratitude” (EN27H71). Nevertheless, half of them differentiate the perception based on whether they are older persons close to them or older people in general: “I think that when they are people close to us it is positive (…) when they are not so close (…) the perception is that when we get older, well, we start being less valued” (EN19MC64). We observe that they mention the relevance of interpersonal contact when they refer to closeness, coexistence, good relationships with older people in one’s immediate environment, even a personal interest in older people as a group: “they have studied social sciences and have a greater appreciation of older people” (EN14H74). 

## 4. Discussion

Older persons are aware of the many examples with which older people, as a group, are negatively portrayed. Vulnerability, the need for protection, their homogeneous condition, the fact that they are not economically productive and imply an expense for society, the devaluation of old age and the burden they entail are stereotypes that have been collected historically in different societies [2,8] and that have also been perpetuated in the context of a pandemic [2,3,4,5,13]. The consideration as a homogeneous group, together with the condition of fragility or vulnerability, have been identified as key factors in the phenomenon of ageism [12]. 

The overprotection targeting them is a matter of concern and we detect that this coincides with the devaluation of their capacity. The literature exposes the phenomenon of compassionate ageism, by which benevolent and positive behaviours are directed towards older people regardless of their desire to be helped [15]. One study [14] showed that older people are object of paternalistic attitudes due to the perception of that group as being warm but not very competent. The attitudes, such as feeling sorry for them, together with the characteristics associated with the older population, as collected in this research, replicate those described in the stereotype content model [14].

The origin of the devaluation of older people, which the interviewees mainly attribute to social changes, coincides with evidence showing that changes in cultural traditions result in changes in the older population’s role, for example, as to the transmission of knowledge [12] and in the prioritisation of certain values over others, such as the youth-centred culture of many societies [18]. Some studies identify the lack of respect by younger generations and have warned of the intergenerational crisis that can arise from the physical or psychological separation of two groups [3,11,23]. Likewise, media information portraying older people unfavourably has been widely documented in the context of the COVID-19 pandemic [5]. 

We consider that the contributions collected about the changes necessary to improve the social imaginary of older people reflect the active role that older people can play in social participation [1]. One study showed that some older people adopt a critical attitude in the face of signs of ageism, for example, by rejecting contents disseminated through information services and communications during the pandemic [2]. As to the reflections of this study’s participants, we observed that the requests to participate in both social activities and employment are consistent with the active ageing approach and the identified need to create societies offering opportunities for the older population’s development [14] [24]. Intergenerational encounters, in turn, are intervention models that have favourably generated mutual recognition and reduced discrimination [1]. In addition, the emphasis on education can facilitate non-stereotyped knowledge of older people [1,20]. 

In this study, the positive image of the older population has been associated with people who remain active and, according to the participants’ words, who remain young until old age. Good physical and health conditions, as well as opportunities for older people to participate in society, are the goal of the active ageing approach that different societies are developing and which the older population may be internalising [14]. The allusion to personal stereotypes that emphasise, above all, the role of older people’s wisdom is a belief coherent with the role traditionally assumed by older people, contrary to the attribution of low capacity expressed by some older adults [2]. For some people, the older population should be considered a reference in the face of social changes, such as the economic and health crisis due to the COVID-19 virus. One study recommended giving older people a leading role in decision making in the context of a pandemic and in the design of the new normality [4]. The study concludes that among the repercussions derived from social changes is a possible variation in the degree to which society manifests ageism [4] and, along the same lines, frames the beliefs that point out that the older adults’ life experience and their resulting coping capacity after crisis periods have possibly led to a more favourable image of the older population. Finally, the differentiation between the attitude towards older people based on whether they are close or not, highlights the importance of intergenerational interventions that bring people together [1,20].

## 5. Conclusions

People over 60 years of age in the Basque Country denounce the stigma of low capacity attributed to older people during the pandemic. They reject the signs of aged-based overprotection shown during the pandemic and describe State measures and social initiatives as paternalist. They highlight the valuation of the knowledge acquired through life experience. They identify older adults as role models for action during crises. They acknowledge that, in general, older people have a better physical appearance until later ages. They express a trend toward valuing older people when they are loved ones. They demonstrate reflection on social problems and offer solutions according to current intervention models. We identified concern about the intergenerational relationship, education based on values and opportunities for the development of active ageing. Given the foregoing, we underline the importance of interventions aimed at reducing the emotional distance between generations, as well as the challenge of making visible the active role that is assumed or may be assumed by older people as a social group. 

This study has limitations in terms of its overrepresentation of women and people with a higher education level. Future qualitative studies could integrate the perspectives of more heterogeneous samples for an in-depth exploration of a larger number of older adults, given the importance of exploring the voices of those individuals who are the protagonists of this process. 

## Data Availability

The data collected in this study can be obtained from the corresponding author upon reasonable request.
